# Modulating Nrf-2/HO-1, apoptosis and oxidative stress signaling pathways by gabapentin ameliorates sepsis-induced acute kidney injury

**DOI:** 10.1007/s00210-023-02650-y

**Published:** 2023-08-07

**Authors:** Mahmoud Abdelnaser, Rania Alaaeldin, Mina Ezzat Attya, Moustafa Fathy

**Affiliations:** 1 Department of Biochemistry, Faculty of Pharmacy, Deraya University, Minia, 61111 Egypt; 2 Department of Pathology, Faculty of Medicine, Minia University, Minia, 61519 Egypt; 3 Department of Biochemistry, Faculty of Pharmacy, Minia University, Minia, 61519 Egypt

**Keywords:** CLP, Gabapentin, Apoptosis, Nrf-2, HO-1, TNF-α, NF-kB, Bax

## Abstract

**Purpose:**

Globally, sepsis, which is a major health issue resulting from severe infection-induced inflammation, is the fifth biggest cause of death. This research aimed to evaluate, for the first time, the molecular effects of gabapentin's possible nephroprotective potential on septic rats by cecal ligation and puncture (CLP).

**Methods:**

Sepsis was produced by CLP in male Wistar rats. Evaluations of histopathology and renal function were conducted. MDA, SOD, GSH, TNF-α, IL-1β, and IL-6 levels were measured. qRT-PCR was utilized to determine the expression of *Bax, Bcl-2*, and *NF-kB* genes. The expression of Nrf-2 and HO-1 proteins was examined by western blotting.

**Results:**

CLP caused acute renal damage, elevated the blood levels of creatinine, BUN, TNF-α, IL-1β, and IL-6, reduced the expression of Nrf-2 and HO-1 proteins and the *Bcl-2* gene expression, and upregulated *NF-kB* and *Bax* genes. Nevertheless, gabapentin dramatically diminished the degree of the biochemical, molecular, and histopathological alterations generated by CLP. Gabapentin reduced the levels of proinflammatory mediators and MDA, improved renal content of GSH and SOD, raised the expression of Nrf-2 and HO-1 proteins and *Bcl-2* gene, and reduced the renal expression of *NF-kB* and *Bax* genes.

**Conclusion:**

Gabapentin mitigated the CLP-induced sepsis-related acute kidney injury through up-regulating Nrf-2/HO-1 pathway, repressing apoptosis, and attenuating the oxidative stress status by reducing the levels of the proinflammatory mediators and enhancing the antioxidant status.

## Introduction

Sepsis, an uncontrolled hyperinflammatory reaction toward infection, is the primary lethal reason in intensive care units (Zarjou and Agarwal 2011). The generation of multiple organ malfunction and systemic inflammatory reactions in critically ill patients due to acute infections is marked by impairments of liver, lung, cardiovascular, renal, and gastrointestinal systems (Singer et al. 2016). One of the most frequent sepsis consequences is acute kidney injury (AKI) (Gonçalves et al. 2010). AKI is developed in about 50% of septic patients, contributing to 70% of septic patient deaths in intensive care units (Höcherl et al. 2010; Lopes et al. 2010). The cecal ligation-puncture (CLP) model closely resembles human sepsis; thus, scientists usually use it to assess the potential protective impacts of many drugs screened for their possible activity against septic shock (Brooks et al. 2014).

Despite the lack of a clear etiology, sepsis-induced AKI has been linked to tubular dysfunction caused by inflammatory responses and oxidative stress, which are aggravated by cytokines release. It is well established that the nuclear factor kappa B (NF-kB) modulates a wide range of genes participating in the innate immune reaction of the body (Fawzy et al. 2021; Zaki et al. 2022). Several inflammatory mediators, such as interleukin-1 beta (IL-1β), interleukin-6 (IL-6), and tumor necrosis factor-alpha (TNF-α), are controlled by NF-kB (Abd El-Baky et al. 2020; Alaaeldin et al. 2022). It has been shown that in polymicrobial septic models, suppressing NF-kB maintains the balance between inflammatory and anti-inflammatory mediators (Brown and Jones 2004; Bitto et al. 2012; Alaaeldin et al. 2023).

The transcriptional factor Nuclear factor erythroid 2- related factor 2 (Nrf-2) supports the cellular defense mechanisms and protects the cells against inflammation and oxidative stress (Vomund et al. 2017). Because of oxidative stress, Nrf-2 secretes an inhibitory protein from its cytoplasm that moves to the nucleus and stimulates many genes implicated in the antioxidant defense mechanism, such as heme oxygenase-1 (HO-1) and superoxide dismutase (SOD) (Vomund et al. 2017; El-Emam et al. 2020). Deficiency of Nrf-2 promotes oxidative stress, which enhances the expression of NF-kB-mediated cytokines, whereas NF-kB is more easily activated in oxidative environments (Yerra et al. 2013).

The structural counterpart of gamma amino butyric acid (GABA), Gabapentin, was prescribed for treating epilepsy for the first time in 1994. It has been shown that Gabapentin modulates calcium influx in nerve terminals by blocking calcium channels, specifically with the α2-δ1 subunit (Maneuf et al. 2006). Thus, Gabapentin regulates several neurotransmitters release, like GABA, noradrenaline, serotonin, glutamate, and substance P (SP) (Cai et al. 2012). It was shown that Gabapentin has analgesic effects in addition to its potent effect on suppressing partial seizures (Gordh et al. 2008). Moreover, it protected rat myocardium from doxorubicin-induced injury by decreasing myocardial caspase-8 and JNK contents (Samra et al. 2021). Moreover, it reduced colitis induced by trinitro benzene sulfonic acid through altering mast cell signaling. It modulated the inflammatory genes participated in the pathogenesis of inflammatory bowel disease by inhibiting their activation and stimulating the peroxisome proliferator-activated receptor gamma (PPAR- γ) (de Brito et al. 2020).

Searching for new pharmacological potentials for natural (Sabra et al. 2022) or synthetic (Shytaj et al. 2022) agents recently attracted attention (Fawzy et al. 2023a). Consequently, for the first time, the current study investigated the possible potential nephroprotective effect of Gabapentin against AKI induced by CLP-evoked sepsis in rats by evaluating Nrf2/ HO-1, apoptosis, and oxidative stress signaling pathways.

## Materials and methods

### Drugs, chemicals, and reagents

By Eipico Pharmaceutical Company (Cairo, Egypt), Gabapentin and vitamin C were supplied. Prior to usage, isotonic saline solution was used to dissolve the drugs.

TRIzol reagent (Life Technologies, United Kingdom, Cat no: 15596026), Cell lysis buffer (Invitrogen, United Kingdom, Cat no: FNN0011), Phosphate-Buffered Saline (10X) pH 7.4 (PBS) (Invitrogen, United Kingdom, Cat no: AM9624), 2-Mercaptoethanol (Life Technologies, United Kingdom Cat no: 35602BID), and Tris-buffered Saline (TBS-T) (Life Technologies, United Kingdom, Cat no: 37535) were utilized.

### Animals

From Minia University, College of Medicine (Minia, Egypt), 120 male Wistar rats (190 ± 10 g) were obtained. They were put in polypropylene cages for a week prior to the experiment, with unrestricted access to conventional laboratory water and food. Following regulations set out by Minia University, College of Pharmacy's Research Ethics Committee, all animal experiments and care were conducted (license number: ES 07/2021).

### Induction of sepsis

As reported, the CLP was utilized as a sepsis model (Ibrahim et al. 2020). Following cleaning with a ten percent povidone-iodine solution and shaving the abdominal wall, xylazine (10 mg/kg) and ketamine (100 mg/kg) were given intraperitoneally (i.p.) to anesthetize rats (Wu et al. 2018). After that, a surgical cut was created in the lower left abdominal region. After cecal exteriorization and ligation with silk surgical suture thread (0.3-mm), two 18-gauge syringe needle punctures were made in the ligated cecal region using an 18-gauge needle. Ligation of the cecum was done at 75% of its whole length. Without CLP, identical procedures were performed on rats who experienced a sham operation.

### Experimental design

Rats were allocated at random into 6 groups (each of 10 rats):

Sham group: Rats were i.p. injected with normal saline (0.5 ml) daily for four days, while the operation was carried out without CLP on day 4.

Gabapentin 100 group: Rats were i.p. injected for four days with 100 mg/kg of Gabapentin.

CLP group: Rats were i.p. injected with normal saline (0.5 ml) daily for four days, while CLP procedure was carried out on day 4.

CLP/Gabapentin 50 group: Rats were i.p. injected for four days with 50 mg/kg of Gabapentin, while CLP procedure was carried out on day 4 (Motavallian et al. 2021).

CLP/Gabapentin 100 group: Rats were i.p. injected for four days with 100 mg/kg of Gabapentin, while CLP procedure was carried out on day 4 (Motavallian et al. 2021).

CLP/vitamin C group: Rats were i.p. injected a single dose of 200 mg/kg of vitamin C on day 4 after CLP procedure (Zhang et al. 2021).

Twenty-four hours following the CLP procedure, all groups experienced animal scarification.

To conduct the survival study, the same previously indicated groups of 60 rats were randomly assigned. The survival rates of all groups were monitored for 10 days.

### Samples collection and tissue preparation

On the fifth day, the rats were given an intraperitoneal injection of 1.6 g/kg of 25% urethane to induce anesthesia (Abdelzaher et al. 2021). Abdominal aortic arteries were accessed to collect the blood samples. After collecting serum for biochemical analysis by centrifugation at 4000 g for 15 min, at -20 °C, the samples were preserved. Rapid dissection and washing in ice-cold (10X) PBS, pH 7.4 was performed on the kidney tissues. Four sample portions of kidney tissue were obtained. The first part was promptly frozen in liquid nitrogen and maintained for further biochemical investigation at -20 °C. For additional western blotting analysis and quantitative real-time PCR (qRT-PCR) examination, the second and third parts were preserved at -80 °C. For histological investigations, the fourth part was finally preserved in formaldehyde (10%).

### Assessment of kidney functions

Following the manufacturer's guidelines, kidney function tests were done using a blood urea nitrogen (BUN) measurement kit (Cat no: 1001331, SPINREACT, Ctra. Santa Coloma, Spain) and a serum creatinine determination kit (Cat no: 11734, Biosystems, Barcelona, Spain).

### Assessment of oxidative stress status

In PBS (10 mM), renal samples were homogenized at pH of 7.4. After mixing the homogenates, they were centrifuged for 10 min at 4000 g at 4 °C. The biochemical analysis required the supernatant, which was collected.

As directed by the manufacturer, the obtained supernatant was tested for the presence of renal reduced glutathione (GSH) (Biodiagnositic, Giza, Egypt, Cat no: GR 2511), malondialdehyde (MDA) (Biodiagnositic, Giza, Egypt, Cat no: MD 2529), and superoxide dismutase (SOD) (Biodiagnositic, Giza, Egypt, Cat no: SD 2521).

### Assessment of serum TNF-α, IL-1β, and IL-6 levels

Inflammatory cytokines in the blood were assessed by ELISA kits specific for rats to detect TNF-α (Elabscience, Texas, United States, Cat no: E-EL-R0019), IL-1β (Elabscience, Texas, United States, Cat no: E-EL-R0012), and IL-6 (Elabscience, Texas, United States, Cat no: E EL R0015).

### Quantitative real-time polymerase chain reaction

A kidney sample weighing 100 mg was ultrasonically homogenized by Branson Digital Sonifier® ultrasonic cell homogenizer (SFX 550, Danbury, Connecticut, United States) with TRIzol reagent (1 mL). Total RNA amount was evaluated, and the purity was assessed as the ratio of A260/A280. qRT-PCR was performed on RNA samples with a purity score of 1.7 or above. Using the Revert Aid First Strand cDNA Synthesis Kit (Thermo fisher scientific, United Kingdom, Cat no: K1622), total RNA (in equal quantities) were transformed into cDNA for every sample. Using single-stranded cDNAs, real-time PCR was performed. Table 1 contains the primer sequences. Using Thermo Scientific Maxima SYBR Green qPCR Master Mix (2X) (Thermo fisher scientific, United Kingdom, Cat no: K0251) and StepOne real-time PCR Detection System (Ref no: 4369074, Applied Biosystems, Singapore) were utilized to produce PCR process. mRNA levels were evaluated by the comparative cycle threshold technique following the adjustment using *GAPDH* as a reference gene.

**Table 1:** Primers' sequences

Genes		Primer sequence
*Bax*	Forward	5'- GGTGTTGACGGTTCACTTGC -3'
	Reverse	5'- AACGCCTGGATGGGCTTTTA -3'
*Bcl-2*	Forward	5'- TGTATCAAACCATGCGGCTG -3'
	Reverse	5'- GGCTGGTTTTACCGCACCTT -3'
*NF-kB*	Forward	5'- TTACGGGAGATGTGAAGATG -3'
	Reverse	5'- ATGATGGCTAAGTGTAGGAC -3'
*GAPDH*	Forward	5'- ACCAACTGCTTAGCCCCCC -3'
	Reverse	5'- GCATGTCAGATCCACAACGG -3'

### Western blotting

Homogenization of renal tissue samples was performed using cell lysis buffer (100 mM NaCl, Triton X-100 buffer (0.5%), EDTA (1 mM), and Tris (20 mM)). For five minutes, protein homogenates (30 μg) were incubated with a loading buffer containing 2-mercaptoethanol before being exposed to sodium dodecyl sulfate–polyacrylamide gel electrophoresis (SDS-PAGE, 12%) at 100 V for two hours.

The Bio-Rad Trans-Blot SD Cell instrument (Bio-Rad, Hercules, California, United States) was used for electroblotting and electrophoresis. Then, for one hour, proteins were blotted onto PVDF membranes and blocked in TBS-T blocking solution containing Tween-20 (0.05%) and non-fat milk (5% w/v). Incubation with primary antibodies, including Anti-Nrf2 antibody (1:1000) (Abcam, Massachusetts, United States, Cat no: ab92946), Anti-HO-1 antibody (1:1000) (Massachusetts, United States, Cat no: ab52947), and β-actin (Santa Cruz Biotechnology, California, United States, Cat no: sc-47778) was performed at 4 °C overnight. In a blocking buffer solution, the secondary antibody horseradish peroxidase-conjugated polyclonal immunoglobulin (1:5000) (Cell Signaling Technologies Inc., Massachusetts, United States, Cat no: 7074) was utilized. The luminescent image analyzer (LAS-4000, Fujifilm Co., Tokyo, Japan) and Chemiluminescence kit (GE Healthcare, Little Chalfont, United Kingdom) were used to identify immunoreactive proteins following the manufacturer's recommendations. Then, The Image Analysis Java (ImageJ, 1.8.0_172) program was utilized to achieve densitometric analysis. After normalizing to the relevant β -actin levels, data were compared to the sham group.

### Histopathological examination

10% neutral buffered formalin solution was used for renal tissue fixation, and the traditional hematoxylin and eosin (H&E) staining was done on kidney tissues (Bancroft and Gamble 2008). Sections were viewed and analyzed by experts in the field with a high-quality digital camera placed on the microscope (Olympus, Tokyo, Japan).

### Statistical analysis

The findings were depicted as mean ± standard deviation (n = 10). ANOVA was performed, proceeded by the Tukey-Kramar post-analytic test. Graph Pad Prism 7 (Graph Pad Software, Boston, MA, United States) was utilized to conduct statistical analyses. The findings were regarded as statistically significant at p-values lower than 0.05.

## Results

### Gabapentin's effect on survival rate of septic rats

**Fig. 1:**
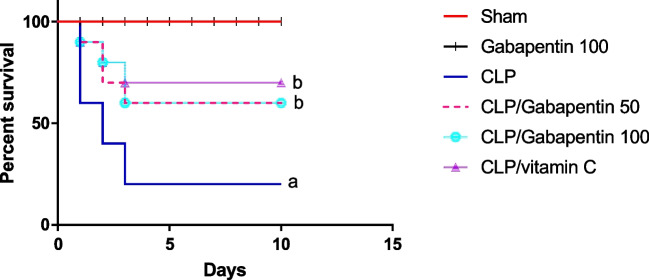
Effect of Gabapentin (50 and 100 mg/kg|) and vitamin C on CLP-caused mortality (n = 10), where a; *p* < *0.05*, by contrast with sham control group, b; *p* < *0.05*, by contrast with CLP group, analyzed by log-rank test CLP: cecal ligation puncture

Figure 1 demonstrates that 40% of the untreated septic rats died within the first day of the operation, in contrast to the sham-operated group, which had no deaths throughout the entire period of observation (10 days). In addition, 60% of the untreated CLP group died by day two. Conversely, the septic groups that received Gabapentin or vitamin C exhibited 10% mortality on the first postoperative day. Furthermore, 40% of the rats died during the overall time of monitoring for Gabapentin treated groups and 30% for vitamin C-treated group. In comparison to the untreated septic group (20%), overall survival was significantly (p < 0.05) higher for both Gabapentin (60%) and vitamin C (70%).

### Gabapentin's effect on kidney functions

**Fig. 2:**
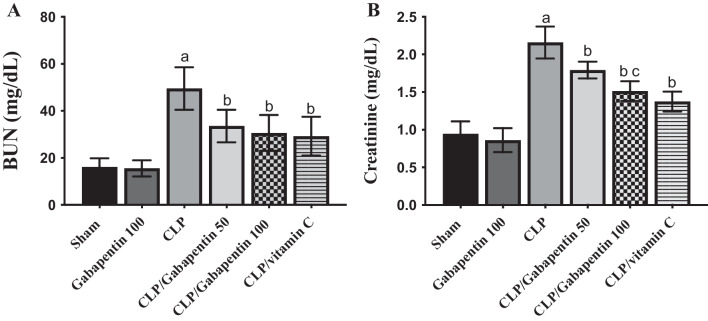
Serum levels of BUN (**A**) and creatinine (**B**). The bars depict mean ± SD. Significant differences were analyzed using one-way ANOVA (n = 10), where a; *p* < *0.05*, by contrast with sham control group, b; *p* < *0.05*, by contrast with CLP group, c; *p* < *0.05*, by contrast with CLP/Gabapentin 50 group. CLP: cecal ligation puncture, BUN: Blood urea nitrogen

Serum BUN besides creatinine concentrations were assessed to appraise the beneficial impact of Gabapentin on CLP-induced sepsis-related AKI. As regard to sham control group, the CLP group demonstrated a marked (P < 0.05) increase in serum creatinine and BUN concentration. The blood concentrations of creatinine and BUN dramatically (P < 0.05) declined in the Gabapentin-treated and vitamin C-treated groups as contrasted to the CLP group. Furthermore, as presented in Fig. 2, the serum creatinine levels of the CLP/Gabapentin 100 group declined notably (P < 0.05) compared to septic rats treated with gabapentin 50 mg.

### Gabapentin's effect on renal SOD, GSH and MDA

**Fig. 3:**
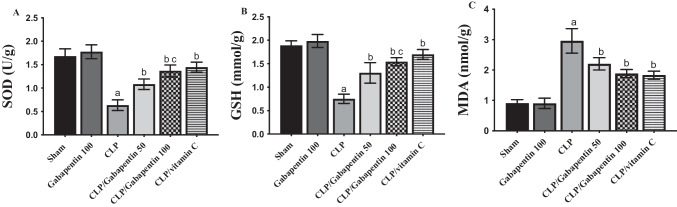
Renal tissue levels of SOD (**A**), GSH (**B**), and MDA (**C**). The bars depict mean ± SD. Significant differences were analyzed using one-way ANOVA (n = 10), where a; *p* < *0.05*, by contrast with sham control group, b; *p* < *0.05*, by contrast with CLP group, c; *p* < *0.05*, by contrast with CLP/Gabapentin 50 group. CLP: cecal ligation-puncture, GSH: reduced glutathione, SOD: superoxide dismutase, MDA: malondialdehyde

Figure 3 depicts SOD, GSH, and MDA levels in the renal tissues of various groups. The renal amounts of GSH and SOD in the CLP group were markedly (P < 0.05) lower, with respect to sham control rats. However, by contrast with the CLP group, treatment with Gabapentin (the two dosages) or vitamin C (the positive control) markedly (P < 0.05) attenuated these alterations. Notably, as regards the low dose of gabapentin, the higher dose significantly (P < 0.05) escalated the GSH and SOD levels.

Also, MDA was considerably (P < 0.05) higher in the CLP group, in comparison to sham control group. Conversely, MDA levels considerably (P < 0.05) dropped in rats treated with Gabapentin and vitamin C, in contrast to CLP group.

### Gabapentin's effect on serum TNF-α, IL-6, and IL-1β levels

**Fig. 4:**
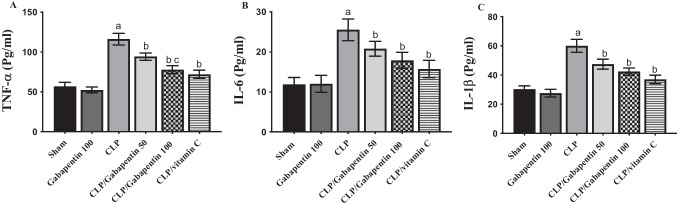
Serum levels of TNFα (**A**), IL-6 (**B**), and IL-1β (**C**). The bars depict mean ± SD. Significant differences were analyzed using one-way ANOVA (n = 10), where a; *p* < *0.05*, by contrast with sham control group, b; *p* < *0.05*, by contrast with CLP group, c; *p* < *0.05*, by contrast with CLP/Gabapentin 50 group. CLP: cecal ligation-puncture, TNF-α: tumor necrosis factor alpha, IL-1β: interleukin 1 beta, IL-6: interleukin 6

After CLP, TNF-α, IL-6, and IL-1β blood levels were markedly (P < 0.05) increased with respect to sham control group, as depicted in Fig. 4. Nevertheless, when rats were given Gabapentin or vitamin C treatments, their blood levels of these cytokines were markedly (P < 0.05) decreased, by contrast with CLP group. Interestingly, TNF-α level was much lower (P < 0.05) in septic rats treated with the high dose of gabapentin, by contrast with the low dose.

### Gabapentin's effect on NF-kB, Bax, and Bcl2 genes expression

CLP group demonstrated a significant (P < 0.05) elevation in mRNA level of renal *NF-kB* expression, by contrast with sham control group. While gabapentin treatment demonstrated considerable (P < 0.05) dose-dependent suppression of renal *NF-kB* gene expression, as opposed to the CLP group.

**Fig. 5:**
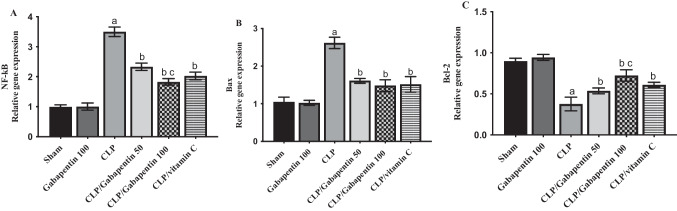
*NF-kB *(**A**),* Bax *(**B**), and *Bcl-2 *(**C**) mRNA levels in renal tissues before and after Gabapentin treatment in CLP-induced sepsis. Quantitative RT-PCR was used to analyze the gene expression of different groups. Expression was compared to the sham control group after being adjusted to that of *GAPDH* gene. The bars depict mean ± SD. *S*ignificant differences* were analyzed using o*ne-way ANOVA *(n* = *10)*, where a; p < 0.05, *by contrast with* sham control group, b; p < 0.05, *by contrast with* CLP group, c; p < 0.05, *by contrast with* CLP/Gabapentin 50 group. Bcl-2: B-cell lymphoma 2, Bax: (Bcl-2)-associated X protein, NF- kB: nuclear factor-kappa B, CLP: Cecal ligation-puncture

Moreover, *Bax* and *Bcl-2* mRNA levels were analyzed to assess the gabapentin's effect on altering factors affecting apoptosis. As illustrated in Fig. 5, with respect to sham control group, CLP dramatically (P < 0.05) elevated the expression of the *Bax* gene and declined (P < 0.05) the expression of the *Bcl-2* gene. However, as contrasted to CLP group, the expression of *Bcl-2* gene was dramatically (P < 0.05) elevated and that of *Bax* was markedly (P < 0.05) decreased in both the Gabapentin-treated septic rats and the vitamin C-treated septic rats.

### Gabapentin's effect on Nrf-2 and HO-1 proteins expression

**Fig. 6:**
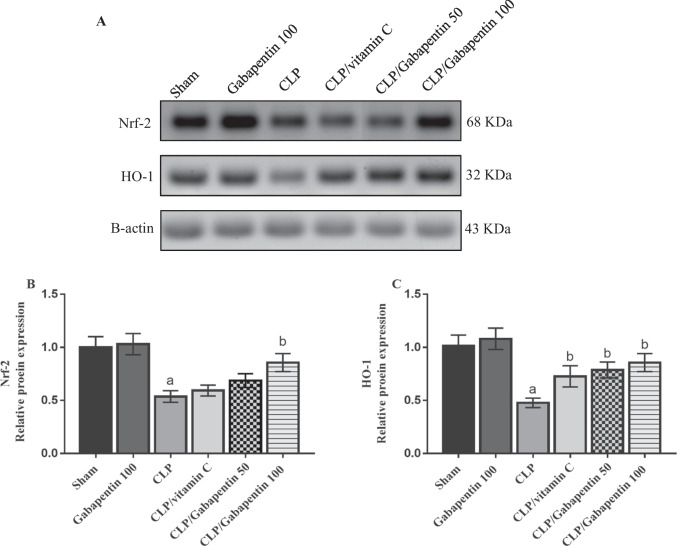
Protein expression of Nrf-2 and HO-1. (**a**) Representative western blots of Nrf-2, HO-1, and β-actin proteins in different groups. (**b & c**) Proteins expressions were determined densitometrically, using bands in (A) after the band intensities have been adjusted to the internal control β-actin, as fold change corresponding to that of sham control rats. The bars depict mean ± SD. One-way ANOVA (n = 3) was used to identify significant differences, where a; p < 0.05, by contrast with sham control group, b; p < 0.05, by contrast with CLP group. HO-1: Heme oxygenase 1, Nrf-2: nuclear factor erythroid 2-related factor 2, CLP: Cecal ligation-puncture

As indicated in Fig. 6, after the band intensities have been adjusted to the internal control β-actin, western blot analysis showed a dramatic (P < 0.05) downregulation of renal Nrf-2 and HO-1 protein of septic untreated rats in comparison to sham control rats. Nevertheless, Gabapentin 100 administration considerably (P < 0.05) upsurged Nrf-2 protein expression by contrast with CLP animals. Additionally, the protein expression of HO-1 was considerably (P < 0.05) increased in the Gabapentin treated septic rats and vitamin C-treated septic rats, in contrast to the CLP group.

### Histopathological results

**Fig. 7:**
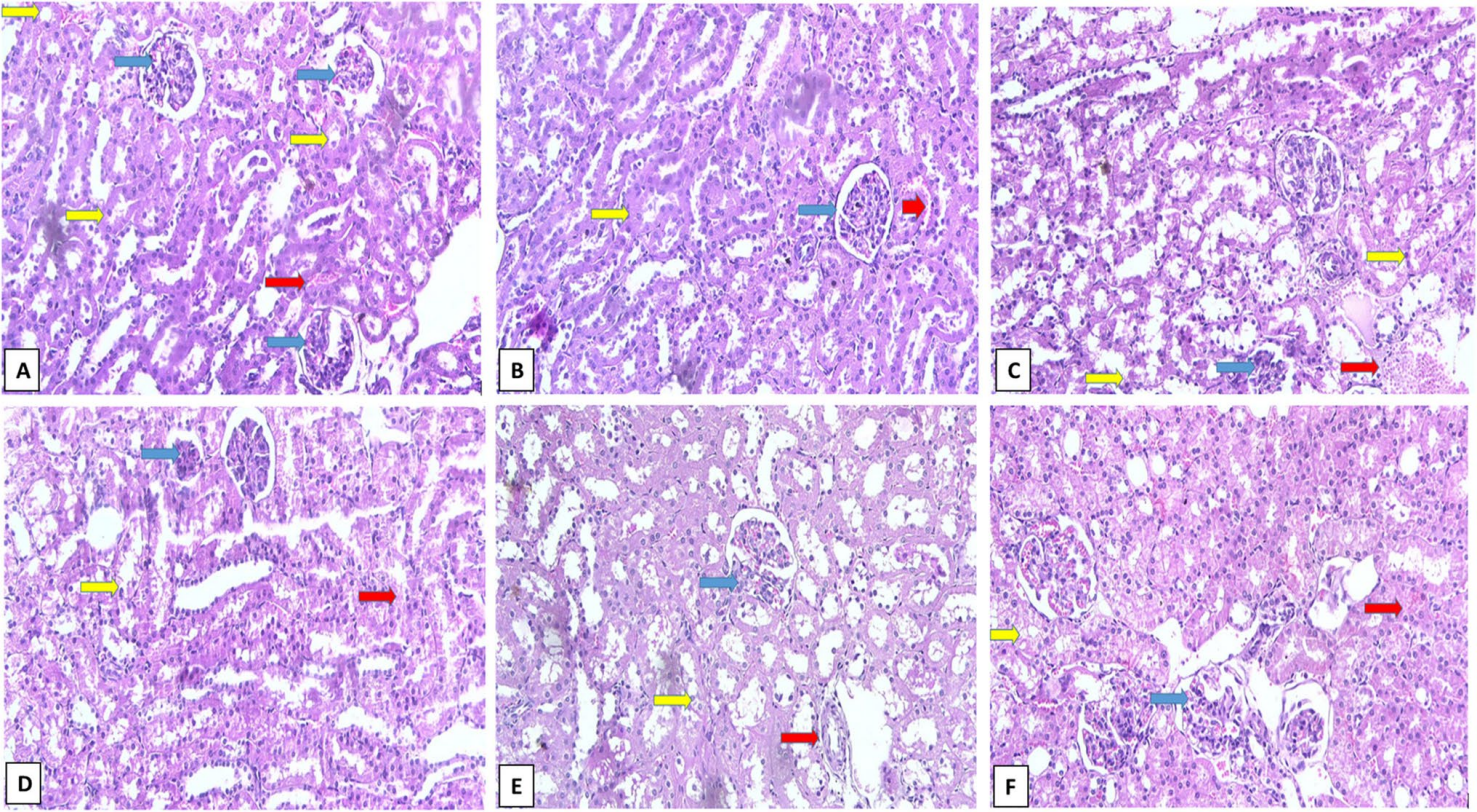
Photomicrographs of rat kidney tissues (H&E staining, × 200) of Sham control group (**A**), Gabapentin 100 mg group (**B**), CLP group (**C**), CLP/Gabapentin 50 (**D**), CLP/Gabapentin 100 (**E**), and CLP/vitamin C (**F**). renal glomeruli (blue arrows), tubules (yellow arrows) and vasculature (red arrow)

As depicted in Fig. 7 and Table 2, CLP group revealed collapsed and necrosed renal glomeruli with widened Bowman’s capsule, degenerated tubules, and areas of interstitial hemorrhage in comparison to sham control group which exhibited normal glomeruli, tubules, and vasculature. Furthermore, CLP/Gabapentin 50 group revealed few necrosed renal glomeruli with widened Bowman’s capsule, tubules showing cloudy swelling, cytoplasmic vacuolations, and areas of interstitial hemorrhage. In addition, CLP/Gabapentin 100 group showed near-normal renal glomeruli, mild tubular cytoplasmic vacuolation, and normal vasculature. However, vitamin C-treated group showed minimal areas of interstitial hemorrhage, mild tubular cytoplasmic vacuolation, and degeneration.

**Table 2:** Scoring representation of renal tissues of different rats

	Sham	Gabapentin 100 mg	CLP	CLP/Gabapentin 50 mg	CLP/Gabapentin 100 mg	CLP/ vitamin C
Interstitial						
1. Inflammatory cells	**-**	**-**	+ +	+ +	+	+
2. Haemorrhage	**-**	**-**	+ + +	+ +	**-**	+
3. Vascular congestion	**-**	**-**	+ + + +	+ + +	-	+
4. Oedema	**-**	**-**	+ + +	+ +	+	+
Renal corpuscle						
1. Widening BC space	**-**	**-**	+ +	+	**-**	+
2. Shrinkage/Distorted corpuscle	**-**	**-**	+ + +	+ +	**-**	+
3. G. vacuolation	**-**	**-**	+ + + +	+ +	+	+
Renal tubules						
1. Cellular vacuolation	**-**	**-**	+ + + +	+ +	+	+ +
2. Cellular apoptosis	**-**	**-**	+ + + +	+ +	+	+
3. Casts	**-**	**-**	+ + +	+	**-**	+ +
4. Lumen widening and distortion	**-**	**-**	+ + +	+	**-**	+

^(−): assigned normal. (+): in between mild and normal level. (++): less than 25% of the total examined fields revealed histopathological alterations (mild level). (+++): less than 50% of the total examined fields revealed histopathological alterations (moderate level). (++++): less than 75% of the total examined fields revealed histopathological alterations (severe level)^

## Discussion

Sepsis is a major global challenge facing medical researchers, physicians, and emergency medicine nowadays due to its complex pathophysiology (Macdonald et al. 2017). AKI is among the highest serious consequences of sepsis, contributing to its increased number of deaths (Gómez and Kellum 2019).

The current study is the first to evaluate the possible potential nephroprotective impact of Gabapentin on CLP-evoked sepsis in rats. Due to the high mortality rate associated with sepsis, we began our investigation by analysing the Gabapentin's effect on the septic rats' survival. Our findings revealed 40% and 80% mortality from sepsis on the first and fourth days, respectively, following surgery. Surprisingly, we demonstrated that early Gabapentin administration before sepsis induction resulted in a significant reduction in mortality to 10% and 40% on the first and fourth days, respectively, following surgery.

The CLP method has been shown to cause sepsis, further renal impairment, and AKI (Yan et al. 2019b). For instance, Luo et al. have documented that resveratrol attenuated the elevated blood levels of creatinine and BUN after sepsis (Luo et al. 2020). In the current research, the concentration of these markers significantly upsurged in the CLP-evoked septic rats. However, they declined considerably in the septic rats treated with Gabapentin, showing that Gabapentin improved kidney function and reduced renal damage.

The severity of sepsis can be increased by oxidative stress and inflammation (Galley 2011; Abdelnaser et al. 2023). As indicators of early mortality in septic rats, SOD and GSH levels were reported by Ritter et al. (Ritter et al. 2003). The current investigation revealed that SOD and GSH, as antioxidant factors, were reduced during the sepsis but dramatically increased, in a dose dependent manner, following the treatment with Gabapentin resulting in promoting the antioxidant status and repressing the oxidative stress condition, suggesting the Gabapentin's renal antioxidant potential of the septic rats. Similarly, previous studies demonstrated that Gabapentin markedly reduced oxidative stress in several organs, including brain, lung, and retina (Abdel-Salam et al. 2012; Yosri et al. 2018; Ola et al. 2019).

Moreover, oxidative stress and apoptosis have been illustrated to be promoted by MDA, which is produced as a result of reactive oxygen species-induced lipid peroxidation (Requena et al. 1996; Fawzy et al. 2022). Our research indicated that the untreated septic rats had significant higher renal MDA levels than the sham group; however, Gabapentin administration significantly lowered these MDA levels, suggesting that Gabapentin may have a role in reducing oxidative stress and attenuating apoptosis against CLP-evoked sepsis.

As a crucial transcription factor, NF-kB regulates the expression of pro-inflammatory cytokines (Surh and Na 2008; Fathy et al. 2019; Abdellatef et al. 2021). Nrf-2 is an essential transcription factor, that modulates the transcription of multiple genes, including HO-1 (Yerra et al. 2013). A deficiency of Nrf-2 promotes oxidative stress, which stimulates the expression of NF-kB-mediated cytokines because NF-kB is more easily activated in an oxidative environment (Li et al. 2008; Fawzy et al. 2023b). Furthermore, it has been reported that the Nrf2 target proteins, phase II detoxifying enzymes and HO-1, control inflammation by inhibiting NF-kB (Park et al. 2015). Sepsis was demonstrated to upsurge the expression of NF-kB and repress Nrf-2 signaling pathway, which further stimulates the expression of TNF-α, IL-1β, and IL-6 (Cai et al. 2017; Wang et al. 2018). Our findings demonstrated that Gabapentin inhibited the expression of TNF-α, IL-1β, and IL-6 by suppressing *NF-kB* gene expression and activating Nrf-2/HO-1 signaling pathway.

The Bcl-2 family is crucial in cells' internal signaling of apoptosis. The Bcl-2 family members, the pro- and anti-apoptotic genes (*Bax* and *Bcl-2*, respectively), have a direct contribution to apoptosis, which is affected by their balance (Chittenden et al. 1995). It has been demonstrated that NF-kB upregulation increased the expression of Bax, the cell death protein, and decreased the expression of the Bcl-2, inducing apoptosis (Zhang et al. 2017; Alaaeldin et al. 2020, 2021). Consequently, the increased Bax/Bcl-2 ratio dramatically promotes apoptosis by changing the structure of the mitochondria and its potential and promoting the apoptotic signalling cascade (Wang et al. 2016). For instance, Lin et al. have demonstrated that fish oil reduced the renal apoptosis induced by CLP by decreasing the expression of Bax/Bcl-2 ratio (Lin et al. 2019).

In accordance with others, our results showed that, in untreated septic rats, the expression of the *Bax* gene was raised. In contrast, the expression of the *Bcl-2* gene was dramatically diminished. More interestingly, in the current work, Gabapentin treatment significantly decreased the expression of the *Bax* gene and markedly elevated the expression of the *Bcl-2* gene, showing its renal anti-apoptotic efficacy during sepsis induced by CLP. Previous studies reported that Gabapentin has anti-apoptotic activity in diabetic rats' retinas and neuronal injury through stimulating Bcl-2 and repressing Bax expression (Ola et al. 2019; Yan et al. 2019a).

In this work, we revealed the underlying molecular mechanisms by which Gabapentin may exert its nephroprotective effects against CLP-induced sepsis-related AKI in rats. Nevertheless, further research and follow-up examinations are needed to understand more molecular pathways through which Gabapentin may exert its beneficial reno-protective effects against CLP-induced sepsis.

## Conclusion

Gabapentin substantially reduced septic-induced renal oxidative stress and inflammation, as shown by the elevation in GSH and SOD levels and the repression in MDA, TNF-α, IL-1β, and IL-6 levels. In addition, it inhibited apoptosis by increasing the expression of *Bcl-2* gene and decreasing the expression of *Bax* gene. Moreover, Gabapentin inhibited the expression of *NF-kB* gene and activated the Nrf-2/HO-1 signaling pathway. Using the CLP-induced sepsis model in rats, we conclude that the promising nephroprotective potential of Gabapentin may be mediated by modulating the Nrf-2/HO-1, oxidative stress, and apoptosis signaling pathways.

## Data Availability

All data generated or analysed during this study are included in this published article.
